# Unraveling the Role of Tumor Necrosis Factor-Alpha in Diabetic Peripheral Neuropathy: A Systematic Review and Meta-Analysis

**DOI:** 10.7759/cureus.49926

**Published:** 2023-12-04

**Authors:** Aniruddha Sen, Palani Selvam Mohanraj, Amit Ranjan, Vinoth Rajendran, Subramaniam ArulVijayaVani, Yuvaraj Balan, Akash Bansal

**Affiliations:** 1 Biochemistry, All India Institute of Medical Sciences, Gorakhpur, Gorakhpur, IND; 2 Physical Medicine and Rehabilitation, All India Institute of Medical Sciences, Gorakhpur, Gorakhpur, IND; 3 Community Medicine & Family Medicine, All India Institute of Medical Sciences, Gorakhpur, Gorakhpur, IND; 4 Biochemistry, Jawaharlal Institute of Postgraduate Medical Education and Research Karaikal, Karaikal, IND; 5 Biochemistry, All India Institute of Medical Sciences, Madurai, Madurai, IND

**Keywords:** tumor necrosis factor-alpha, meta-analysis, systematic review, diabetes mellitus, diabetic peripheral neuropathy

## Abstract

Diabetic peripheral neuropathy (DPN) is a prevalent and debilitating complication of diabetes mellitus, leading to sensory abnormalities, decreased balance, and increased risk of foot problems. Although tumor necrosis factor-alpha (TNF-α) has emerged as a potential factor in the pathogenesis of DPN, its role remains contested. This study intends to thoroughly analyze the association between TNF-α and DPN by combining data from various global studies. This systematic review and meta-analysis adhered to the Preferred Reporting Items for Systematic Reviews and Meta-Analyses guidelines and included 23 articles investigating TNF-α levels in DPN patients for systematic review and 11 articles for meta-analysis. Data were extracted, and heterogeneity was examined. A random-effect model was chosen due to high heterogeneity. The major outcome measure across studies was serum TNF-α levels. The meta-analysis found a significant mean difference of 15.2464 (95% confidence interval = 4.4963; 25.9965) under the random-effect model due to the substantial heterogeneity (I^2^ = 98.1%) among included studies. The meta-analysis indicates a consistent elevation in TNF-α levels in individuals with DPN compared to those without neuropathy. This underlines the potential of TNF-α as a biomarker and contributor to diabetic neuropathy. Despite heterogeneity, the study’s extensive scope and systematic approach enhance the trustworthiness and generalizability of the findings.

## Introduction and background

Diabetic peripheral neuropathy (DPN) is a prevalent and often under-recognized consequence of diabetes mellitus. It often presents as sensory abnormalities, including numbness, lack of sensation, and decreased balance, which can greatly reduce a patient’s quality of life. Moreover, DPN raises the risk of foot ulcers and, in extreme situations, may lead to amputation [1]. In India alone, a survey conducted in 2020 indicated that 26-31% of diabetes patients get DPN, with a rising frequency of amputations linked to the illness [2]. The American Diabetes Association has released recommendations for diabetic foot care to address these concerns [3].

In the convoluted pathophysiology of DPN, there is a rising interest in the role of tumor necrosis factor-alpha (TNF-α), a versatile cytokine with significance in inflammatory responses. While factors such as glucotoxicity and the production of advanced glycated end products have been implicated in the neurotoxic pathogenesis of diabetes, the significance of TNF-α in neurotoxic consequences cannot be overlooked [4]. TNF-α, previously identified for its immunological and inflammatory effects, is now being recognized to play a major role in the complicated pathways contributing to neuropathic problems in diabetes [5]. Several inflammatory, free radicals, antioxidant, coagulation, and lipid peroxidation markers have been studied concerning DPN but have not proven helpful as standalone biomarkers. Numerous studies have investigated the potential link between higher TNF-α levels and the development and progression of DPN [6-9]. Inflammation, a hallmark of diabetes-induced problems, is strongly linked with the neurodegenerative processes identified in DPN. TNF-α, as a proinflammatory cytokine, is implicated in the beginning and persistence of inflammatory cascades that lead to nerve injury, demyelination, and decreased nerve conduction [5].

The significance of TNF-α in DPN is a subject of substantial study and controversy, with some studies proposing it as a potential marker for early diagnosis and others exploring its therapeutic potential as a target for intervention. However, the existing literature on TNF-α and DPN shows various findings and interpretations, leading to inconsistent conclusions. To address this diversity and provide a thorough perspective, this systematic review and meta-analysis aims to synthesize and critically examine studies of various study designs to present a comprehensive overview of the connection between TNF-α and DPN.

## Review

Methodology

The systematic review followed the guidelines outlined by Preferred Reporting Items for Systematic Reviews and Meta-Analyses (PRISMA), a framework designed to facilitate the reporting of systematic reviews and meta-analyses [10]. The protocol for the review was registered on PROSPERO (registration ID: CRD42023441858) before the commencement of the study.

Selection Procedure

The systematic review aimed to investigate the role of TNF-α in DPN. Eligible study designs included studies exploring the role of TNF-α in the development, progression, or management of DPN, with no specific restrictions on study types. The participants of the included study were individuals with DPN. The exposure under scrutiny was TNF-α levels, with a comparator group consisting of individuals with diabetes mellitus without neuropathy or healthy volunteers. The study context encompassed settings relevant to understanding the implications of TNF-α in DPN. The primary outcomes of the included study involved assessing neuropathy severity through nerve conduction studies or neurological examination scores.

Search Strategy

We conducted a systematic search of the literature on PubMed, Scopus, Google Scholar, and CINAHL from their inception until July 2023. Furthermore, we delved into the bibliographies of relevant articles for additional insights. Our inclusion criteria focused on studies published exclusively in the English language, employing the subsequent search strategy depicted in Table 1.

**Table 1:** Search strategy. CINAHL: Cumulative Index to Nursing and Allied Health Literature

Database	Search terms
PubMed	(“diabetic neuropathies”[MeSH Terms] OR (“diabetic”[All Fields] AND “neuropathies”[All Fields]) OR “diabetic neuropathies”[All Fields]) AND (“tumour necrosis factor alpha”[All Fields] OR “tumor necrosis factor alpha”[MeSH Terms] OR (“tumor”[All Fields] AND “necrosis”[All Fields] AND “factor alpha”[All Fields]) OR “tumor necrosis factor alpha”[All Fields] OR (“tumor”[All Fields] AND “necrosis”[All Fields] AND “factor”[All Fields] AND “alpha”[All Fields]) OR “tumor necrosis factor alpha”[All Fields] OR (“tumor necrosis factor alpha”[MeSH Terms] OR (“tumor”[All Fields] AND “necrosis”[All Fields] AND “factor alpha”[All Fields]) OR “tumor necrosis factor alpha”[All Fields] OR (“tnf”[All Fields] AND “alpha”[All Fields]) OR “tnf alpha”[All Fields]))
Scopus	TITLE-ABS-KEY ((diabetic AND neuropathies) AND ((tumor AND necrosis AND factor AND alpha) OR (TNF AND alpha)))
Google Scholar	allintitle: Diabetic Neuropathy “Tumor necrosis factor” OR TNF
CINAHL	Boolean/Phrase: Diabetic Neuropathy AND Tumor necrosis factor; Expanders: Apply equivalent subjects; Language: English

Screening and Data Analysis

Studies identified underwent initial screening based on their titles and abstracts by two independent authors. Those deemed potentially relevant underwent further scrutiny, with full texts reviewed by two independent authors using the open-access online tool CADIMA version 2.2.3 from the Julius Kühn Institute in Quedlinburg, Germany. Any disagreements were resolved through discussion or consultation with a third author. Data extraction from selected studies was performed independently by two authors using a standardized form, with discrepancies resolved through consensus or consultation with another author. The extracted data encompassed participant demographics, sample characteristics, exposure details, and study specifics, all recorded in an Excel spreadsheet. R statistical software was employed for data analysis, with mean difference (MD) and 95% confidence intervals (CIs) expressed for continuous variables using inverse variance methods. Heterogeneity among studies was assessed through Cochran’s Q and I² statistics, guiding the choice between fixed-effect or random-effect models based on heterogeneity.

Quality Assessment

**Figure 1:**
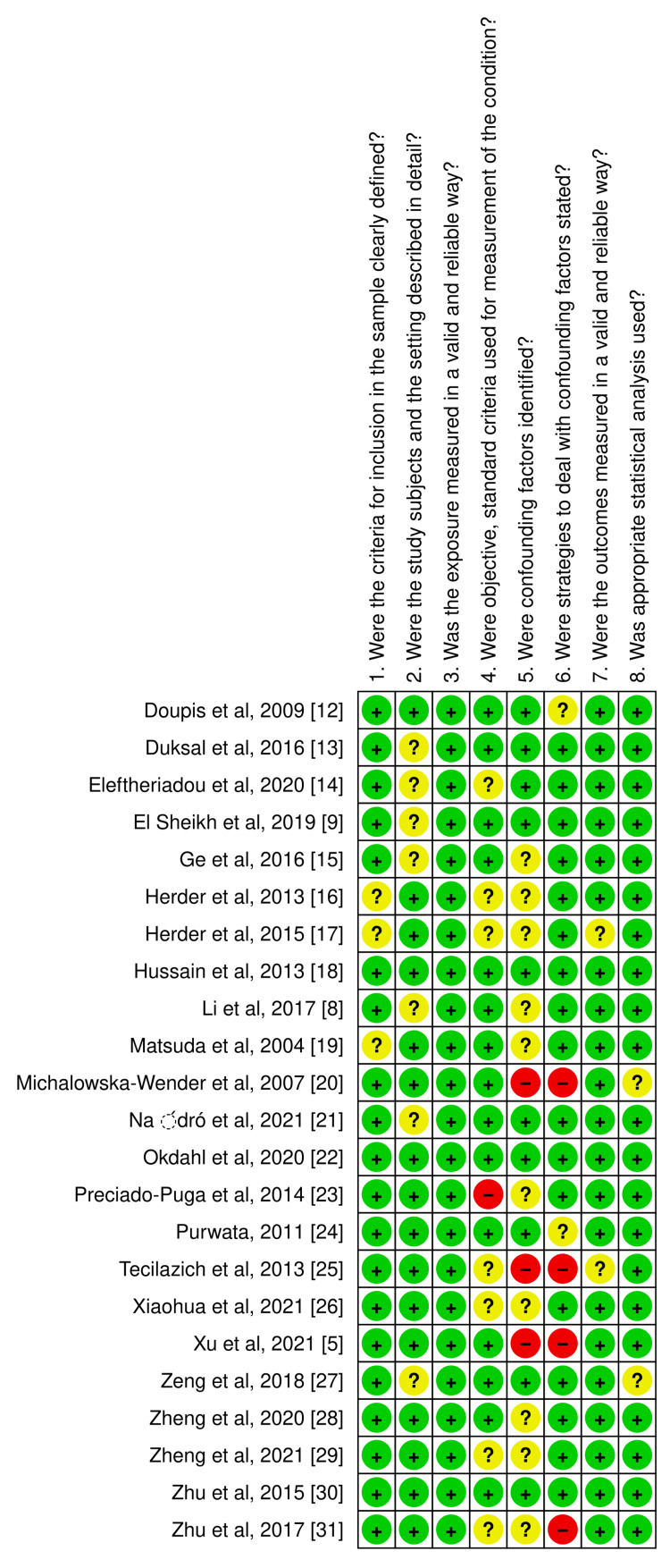
Quality assessment graph of the included studies using the critical appraisal checklist for cross-sectional studies by the Joanna Briggs Institute.

**Figure 2:**
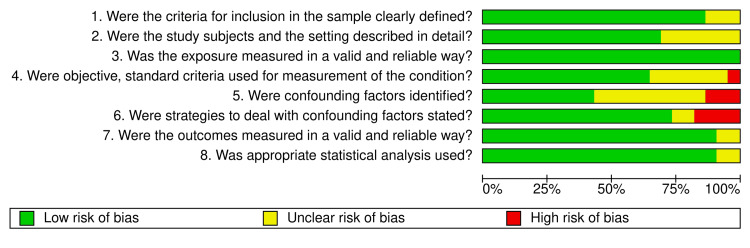
Quality assessment summary of the included studies using the critical appraisal checklist for cross-sectional studies by the Joanna Briggs Institute.

Quality assessment and identification of biases in the reviewed studies were performed using the critical appraisal checklist for cross-sectional studies by the Joanna Briggs Institute [11], and the quality assessment graph (Figure 1) and summary (Figure 2) was generated using RevMan 5.4 from the Cochrane Collaboration.

Results

**Figure 3:**
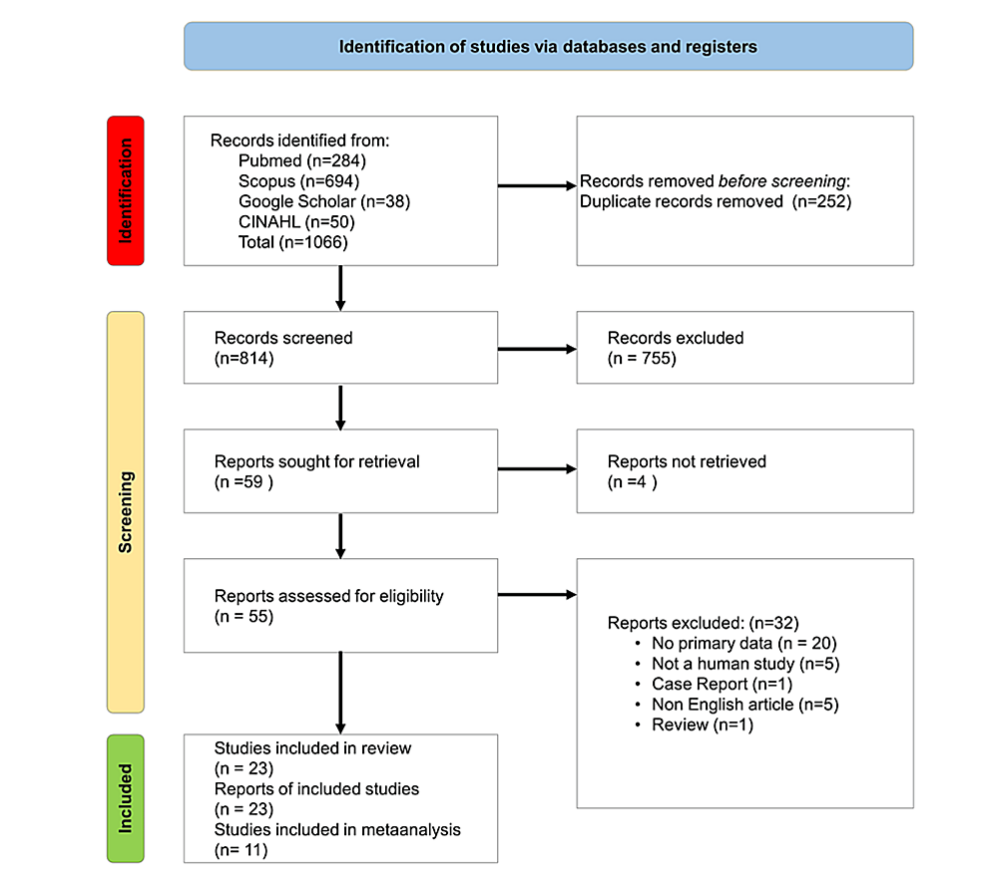
Preferred Reporting Items for Systematic Reviews and Meta-Analyses (PRISMA) flow diagram.

The search strategy initially identified 1,066 articles, distributed across PubMed (284), Scopus (694), Google Scholar (38), and CINAHL (50). After eliminating duplicates and consolidating results, a total of 818 articles were identified. The PRISMA flowchart in Figure 3 outlines the search process. Subsequent screening of titles and abstracts narrowed down the selection to 63 articles for full-text screening. Nine reports were unretrievable, and 31 were excluded for reasons detailed in Figure 3. Ultimately, 23 articles were included in the systematic review, with 11 featuring TNF-α expressed as MD selected for meta-analysis. The studies, conducted in 13 countries globally, demonstrated true diversity, primarily focusing on DPN patients, with sample sizes ranging from 50 to 483 patients. The main outcome measure across studies was serum TNF-α levels.

Table 2 presents a complete overview of studies examining DPN in varied groups. Studies ranged throughout the United States, Turkey, Egypt, Greece, China, Germany, India, Japan, Hungary, Denmark, Mexico, and Indonesia, and involved both type 2 and type 1 diabetes patients, prediabetic individuals, and healthy controls. Duration of diabetes varied, with major variances in the measures adopted for assessment. Common measures included the Neuropathy Symptom Score (NSS), Neuropathy Disability Score (NDS), nerve conduction investigations, clinical examinations, and specialized scoring systems such as the Toronto Clinical Scoring System (TCSS) and Michigan Neuropathy Screening Instrument (MNSI).

**Table 2:** Basic characteristics of the included studies. *: Data are presented as mean ± SD or median (range). DPN: diabetic peripheral neuropathy; T2DM: type 2 diabetes mellitus; NCS: nerve conduction studies; NSS: Neuropathy Symptom Score (NSS); NDS: Neuropathy Disability Score; MNSI: Michigan Neuropathy Screening Instrument; TCSS: Toronto Clinical Scoring System; CPT: current perception threshold; DN4: Douleur Neuropathique 4 questionnaire; KORA: Cooperative Health Research in the Region of Augsburg (German study); PAD: peripheral artery disease

Study	Country	Population	Duration of diabetes	Measures of DPN
Doupis et al., 2009 [12]	United States	T2DM patients with and without DPN	14 ± 11 years in the non-DPN group, and 20 ± 13 years in the DPN group	NSS and NDS
Duksal et al., 2016 [13]	Turkey	Patients with prediabetes, T2DM, and healthy controls	Mean 1.13 years in T2DM	NSS, NDS, and NCS
Eleftheriadou et al., 2020 [14]	Greece	T2DM patients with and without DPN	Median 12.5 years in the DPN group, and 13 years in the non-DPN group	NSS and NDS
El Sheikh et al., 2019 [9]	Egypt	T2DM patients with and without DPN	Not reported	NSS, NDS, and NCS
Ge et al., 2016 [15]	China	T2DM patients with and without DPN	Not reported	NCS
Herder et al., 2013 [16]	Germany	People with T2DM aged 61–82 years from the population-based KORA F4 study	Not reported	Clinical examination; MNSI score
Herder et al., 2015 [17]	Germany	People with T2DM aged 61–82 years from the population-based KORA F4 study	Median 3 years in the non-DPN group, and 8 years in the DPN group	Bilateral impairment of foot vibration perception and/or foot pressure sensation
Hussain et al., 2013 [18]	India	T2DM patients with and without DPN	Not reported	NSS, NDS, and NCS
Li et al., 2017 [8]	China	Patients with impaired glucose regulation, with and without peripheral neuropathy	Not reported	NCS and TCSS
Matsuda et al., 2004 [19]	Japan	T2DM patients	Mean 10.4 years in T2DM	NCS
Michałowska-Wender et al., 2007 [20]	Poland	Patients with diabetic and alcoholic polyneuropathy	Not reported for the DPN group	NCS
Na ´dró et al., 2021 [21]	Hungary	T2DM patients with peripheral neuropathy	Mean 12.4 years	CPT, Ewing’s cardiovascular reflex tests, and DN4 questionnaire
Okdahl et al., 2020 [22]	Denmark	Type 1 diabetes patients with and without DPN and healthy controls	Median 20 years in the non-DPN group, and 32.4 years in the DPN group	NCS and quantitative sensory testing
Preciado-Puga et al., 2014 [23]	Mexico	Patients with T2DM	Mean 11 years in T2DM	Michigan Diabetic Neuropathy Score
Purwata, 2011 [24]	Indonesia	T2DM patients with and without painful DPN	5.30 ± 3.05 years	NCS
Tecilazich et al., 2013 [25]	USA	Patients with T2DM with and without lower extremity complications (neuropathy, PAD) and healthy controls	Not reported for overall population. Mean 12 years in T2DM without complications	Clinical examination, NDS, and quantitative sensory testing
Xiaohua et al., 2021 [26]	China	Patients with T2DM	Not reported	MNSI score
Xu et al., 2021 [5]	China	T2DM patients with and without DPN	5.33 ± 1.79 years in the non-DPN group, 7.34 ± 1.99 years in the DPN group	Symptoms, physical examination, electrophysiology
Zeng et al., 2018 [27]	China	Patients with prediabetes, diabetes and healthy controls	Mean 1.24 years in diabetes group	NSS, NDS, and NCS
Zheng et al., 2020 [28]	China	T2DM patients with and without DPN	Not reported	TCSS and electromyography
Zheng et al., 2021 [29]	China	T2DM patients with and without DPN at baseline	Not reported for the whole cohort, 15.04 ± 10.26 years in the DPN group vs. 12.35 ± 8.97 years in the non-DPN group	MNSI score
Zhu et al., 2015 [30]	China	T2DM patients with and without DPN and healthy controls	Not reported	NCS and neurologic examination
Zhu et al., 2017 [31]	China	Healthy controls and T2DM patients with and without DPN	Mean 7.5 years in T2DM, 10.8 years in the DPN group	Not clearly reported

Table 3 provides a complete summary of sample sizes, mean ages, gender distributions, and study designs. The study designs ranged from cross-sectional, case-control, to longitudinal, demonstrating the multidimensional approach to understanding DPN across different groups and circumstances.

**Table 3:** Details of the included studies. DPN: diabetic peripheral neuropathy; T2DM: type 2 diabetes mellitus; M: males; F: females; IGR: impaired glucose regulation; PN: peripheral neuropathy; PAD: peripheral artery disease

Study	Sample size	Mean age, years	Gender distribution	Study design
Doupis et al., 2009 [12]	212 patients - 55 controls, 80 non-DPN, 77 DPN (31 painless, 46 painful)	55 ± 13 years for controls; 56 ± 14 years for non-DPN; 58 ± 9 years for DPN	56% males in the control group; 55% males in the diabetic non-neuropathy group; 66% males in the diabetic neuropathy group	A cross-sectional study
Duksal et al., 2016 [13]	50 prediabetes, 50 T2DM, and 44 controls	52–53 years	More females than males	A cross-sectional controlled study
Eleftheriadou et al., 2020 [14]	50 patients with DPN, 30 without DPN, and 25 healthy controls	62.5 years in the DPN group, 61.1 years in the non-DPN group, and 61.7 years in controls	24 M/26 F in the DPN group, 16 M/14 F in the non-DPN group, and 9 M/16 F in controls	A cross-sectional study
El Sheikh et al., 2019 [9]	120 patients - 40 non-DPN, 40 mild DPN, 40 moderate-severe DPN	55.9 ± 8.2 years in the mild DPN group, 59.3 ± 7.7 years in the moderate-severe DPN group	74 males, 46 females	A cross-sectional study
Ge et al., 2016 [15]	178 DPN, 87 non-DPN, and 101 controls	Around 53 years in the DPN group, 52 years in other groups	More males in all groups	A case-control study
Herder et al., 2013 [16]	1,047	70.5 years	49% males	A cross-sectional study
Herder et al., 2015 [17]	215 (168 non- DPN, 47 DPN)	71.6 years in the non-DPN group, and 71.8 years in the DPN group	59% males in the non-DPN group, and 66% males in the DPN group	A cross-sectional study
Hussain et al., 2013 [18]	86 (37 with short-duration DPN, 27 with long-duration DPN, 22 without DPN)	Not reported	Not reported	A cross-sectional study
Li et al., 2017 [8]	70 patients with IGR (30 with PN, 40 without PN), and 40 healthy controls	Around 59 years in the IGR-PN group, 58 years in the control group	Equal number of males and females in each group	A cross-sectional study
Matsuda et al., 2004 [19]	105	60.8 years	66 M/39 F	A cross-sectional study
Michałowska-Wender et al., 2007 [20]	29 DPN, 31 with alcoholic neuropathy, and 20 controls	48.3 years in the diabetic group, 54.5 years in the alcoholic group, and 49.1 years in controls	21 M/8 F in the diabetic group, and 23 M/8 F in the alcoholic group	A case-control study
Nádró et al., 2021 [21]	54 DPN, 24 Non-DPN	64.1 years in the DPN group, and 63.58 years in the non-DPN group	22 M, 32 F in the neuropathy group; 11 M, and 13 F in the control group	A prospective study
Okdahl et al., 2020 [22]	50 DPN, 53 non DPN, and 21 controls	Around 51 years in all groups	More females in the non-DPN group, and more males in the DPN groups	A cross-sectional study
Preciado-Puga et al., 2014 [23]	157 at baseline, and 142 at one year	52 years	110 F, 47 M	Longitudinal study with a one-year follow-up
Purwata, 2011 [24]	59 with painful DPN, and 51 without pain	54.1 years	61 F, 49 M	A cross-sectional and case-control study
Tecilazich et al., 2013 [25]	42 (14 controls, 11 non-DPN, 10 DPN, and 7 T2DM with neuropathy and PAD)	57 years in controls, 63 years in the non-DPN group, 63 years in the DPN group, 66 years in T2DM neuropathy and PAD	57% males in controls, 55% males in the non-DPN group, 40% males in the DPN group, 71% males in T2DM neuropathy + PAD	A cross-sectional study
Xiaohua et al., 2021 [26]	483 (86 non-DPN, 176 painless DPN, 221 painful DPN)	54 years in non-DPN, 54.5 years in painless DPN, and 54 years in painful DPN	49% males in the non-DPN group, 101 males in the painless DPN group, and 135 males in the painful DPN group	A cross-sectional study
Xu et al., 2021 [5]	169 (44 non-DPN, 83 DPN, 42 controls)	54.07 ± 7.76 years in the non-DPN group, 56.17 ± 6.28 years in the DPN group, 53.98 ± 5.50 years in controls	24 M/20 F in the non-DPN group, 44 M/41 F in the DPN group, and 22 M/18 F in controls	A case-control study
Zeng et al., 2018 [27]	55 prediabetes, 55 diabetes, and 48 controls	Around 52 years in all groups	Not reported	A case-control study
Zheng et al., 2020 [28]	37 non-DPN, 29 mild DPN, and 15 moderate-severe DPN	58.13 ± 12.63 years overall	47 M, 34 F	A cross-sectional study
Zheng et al., 2021 [29]	315 patients, 106 with blood samples analyzed	63.7 years in the non-DPN group, 66.9 years in the DPN group	Not reported	A rospective cohort study with a five-year follow-up
Zhu et al., 2015 [30]	32 DPN patients, 32 non-DPN, and 30 healthy controls	56 in the DPN group, 54.4 in the non-DPN, 57.4 in controls	Matched between groups	A cross-sectional study
Zhu et al., 2017 [31]	19 controls, 18 non-DPN, and 20 DPN	57–58 years	Approximately equal males and females	A cross-sectional study

Table 4 combines findings from numerous studies on the association between TNF-α levels and DPN. Doupis et al. (2009) [12] demonstrated higher TNF-α in DPN patients, specifically those with painful symptoms. Duksal et al. (2016) [13] observed a positive connection between TNF-α and neuropathy severity. El Sheikh et al. (2019) [9] found increased TNF-α in mild and moderate-severe DPN compared to no DPN. Conversely, Eleftheriadou et al. (2020) [14] observed no significant TNF-α difference between diabetics with and without DPN. Ge et al. (2016) [15] related TNF-α with DPN development. Herder et al (2013) [16] and (2015) [17] demonstrated no significant correlation with DSPN or omentin levels but suggested a potential link with the presence of neuropathy. Studies by Hussain et al. (2013) [18], Li et al. (2017) [8], Matsuda et al. (2004) [19], and Nádró et al. (2021) [21] revealed greater TNF-α in DPN patients, corresponding with decreased nerve conduction velocities and increased peripheral neuropathy risk. Okdahl et al. (2020) [22] observed increased TNF-α in DPN, associated with neuropathy severity. Preciado-Puga et al. (2014) [23] revealed TNF-α predicting complication progression. Purwata (2011) [24] correlated increased TNF-α with uncomfortable DPN and pain severity. Xiaohua et al. (2021) [25] connected TNF-α to painful DPN and vitamin D insufficiency. Xu et al. (2021) [5] demonstrated increased TNF-α in DPN, positively linked with apelin levels. Studies by Zeng et al. (2018) [27], Zheng et al. (2020) [28], Zheng et al. (2021) [29], Zhu et al. (2015) [30], and Zhu et al. (2017) [31] repeatedly indicated higher TNF-α in DPN patients, underlining its potential function as a biomarker and contributor to diabetic neuropathy.

**Table 4:** Details of the interventions used and study outcomes. TLR4: toll-like receptor 4; TNF: tumor necrosis factor; DPN: diabetic peripheral neuropathy; ICAM-1: intercellular adhesion molecule 1; T2DM: type 2 diabetes mellitus; IL-6: interleukin 6; PAD: peripheral artery disease; AGE: advanced glycation end-product; ALA: alpha-lipoic acid; PGRN: progranulin; CPT: current perception threshold; GRO: growth-regulated oncogene; DSPN: diabetic sensorimotor polyneuropathy; MNSI: Michigan Neuropathy Screening Instrument; EPC: endothelial progenitor cells; SDF-1: stromal cell-derived factor 1

Study	Comparison group	TNF-α level	Outcome	Dropout
Doupis et al., 2009 [12]	Controls vs. non-DPN vs. DPN (painful vs. painless)	Higher in the DPN group (7.1 pg/mL) vs. the non-DPN group (5.3 pg/mL) and controls (4.7 pg/mL)	Higher inflammatory cytokines including TNF-α in DPN patients. Further increase in patients with painful DPN	Not reported
Duksal et al., 2016 [13]	Healthy controls	Significantly higher in type 2 diabetes patients vs. controls. No significant difference between prediabetic patients and controls	TNF-α positively correlated with the severity of neuropathy. No correlation between TNF-α and nerve conduction abnormalities	Not reported
Eleftheriadou et al., 2020 [14]	Diabetics without DPN and healthy controls	No significant difference between groups	EPC levels increased in the DPN group vs. non-DPN and controls. SDF-1 levels decreased in the DPN group vs. controls	Not reported
El Sheikh et al., 2019 [9]	Non-DPN vs. mild DPN vs. moderate-severe DPN	Higher in mild DPN (55.22 ± 12.80 pg/mL) and moderate-severe DPN (77.31 ± 19.31 pg/m:) vs. non-DPN (27.26 ± 16.66 pg/mL)	Higher TNF-α levels in DPN patients. Negative correlation between TNF-α and nerve conduction velocity	Not reported
Ge et al., 2016 [15]	Diabetics without DPN and healthy controls	Higher in DPN vs. other groups	TNF-α was associated with DPN development. Positively correlated with anti-ganglioside antibodies	Not reported
Herder et al., 2013 [16]	Subjects with vs. without clinical DSPN; MNSI score ≤2 vs. >2	No significant association between TNF-α and DSPN or MNSI scores	Serum TNF-α levels were not associated with clinical DSPN or MNSI scores	Not reported
Herder et al., 2015 [17]	Non-DPN vs. clinical DPN	Inverse correlation between omentin and TNF-α (r = -0.30, p = 0.019). No significant difference in TNF-α levels between groups	Lower serum omentin levels were associated with the presence of DPN, independent of risk factors and biomarkers of inflammation such as TNF-α	Not reported
Hussain et al., 2013 [18]	Short-duration DPN, long-duration DPN, and non-DPN	Raised in the DPN groups compared to the non-DPN group	Higher serum TNF-α levels in DPN patients, correlated with lower nerve conduction velocities	Not reported
Li et al., 2017 [8]	IGR patients without PN and healthy controls	Significantly higher in the IGR-PN group vs. the IGR-NPN group and controls	TNF-α was an independent risk factor for peripheral neuropathy in IGR patients. Correlated with neuropathy severity	Not reported
Matsuda et al., 2004 [19]	None	Negatively correlated with sensory nerve conduction velocity	TNF-α contributed to sensory neuropathy in type 2 diabetes patients	Not reported
Michałowska-Wender et al., 2007 [20]	Healthy controls	No significant difference between groups	GRO-α levels were higher in both polyneuropathy groups vs. controls. May contribute to myelin lesions	Not reported
Na ´dró et al., 2021 [21]	Diabetic without neuropathy	Significantly higher in the neuropathy group vs. controls at baseline. Decreased after ALA treatment	ALA treatment increased serum PGRN levels and improved CPT values in neuropathy patients. PGRN was positively correlated with inflammatory markers	Not reported
Okdahl et al., 2020 [22]	Type 1 diabetes without DPN and healthy controls	Higher in DPN vs. non-DPN	Multiple inflammatory markers including TNF-α were higher in DPN and associated with neuropathy severity	Not reported
Preciado-Puga et al., 2014 [23]	None, stratified by severity of complications	Higher in those with progression of complications at one year	TNF-α predicted complication progression. AGEs increased with renal dysfunction	15 patients were lost to follow-up
Purwata, 2011 [24]	Painful DPN vs. painless DPN	Higher in painful DPN (17.44 ± 8.23 pg/mL) vs. painless DPN (12.30 ± 3.76 pg/mL)	Higher TNF-α levels in painful DPN patients. Positive correlation between TNF-α and pain severity.	Not reported
Tecilazich et al., 2013 [25]	T2DM without complications vs. with neuropathy vs. with neuropathy + PAD	Higher in T2DM neuropathy and neuropathy and PAD groups compared to controls and T2DM without complications. Associated with impaired mitochondrial function	Impaired mitochondrial oxidative phosphorylation in T2DM with neuropathy, with or without PAD, associated with increased TNF-α and inflammation	Not reported
Xiaohua et al., 2021 [26]	No DPN vs. painless DPN vs. painful DPN	Higher in painful DPN group compared to non-DPN and painless DPN groups. Independent risk factor for painful DPN	Severe vitamin D deficiency associated with increased TNF-α and IL-6 levels in painful DPN group.	Not reported
Xu et al., 2021 [5]	Non-DPN vs. DPN vs. healthy controls	Higher in DPN vs. non-DPN and controls	Higher apelin levels associated with the presence of DPN. Decreased after treatment. Positively correlated with TNF-α	Two in the DPN treatment group
Zeng et al., 2018 [27]	Healthy controls	Higher in diabetes vs. prediabetes and controls. No difference between prediabetes and controls	TNF-α positively correlated with neuropathy severity. Levels differed in patients with vs. without neuropathy	Not reported
Zheng et al., 2020 [28]	No DPN vs. mild DPN vs. moderate-severe DPN	Higher in mild DPN (85.6 pg/mL) and moderate-severe DPN (76.0 pg/mL) vs. non-DPN (35.7 pg/mL)	Higher levels of inflammatory cytokines including TNF-α in patients with DPN. Positive correlation between cytokines and negative emotions	Not reported
Zheng et al., 2021 [29]	Non-DPN group vs. DPN group	Higher in DPN group (3.15 pg/mL) vs. non-DPN group (2.45 pg/mL) at baseline	63 out of 106 patients developed DPN after 5 years. Higher TNF-α and ICAM-1 levels predicted incidence of DPN	Not reported
Zhu et al., 2015 [30]	T2DM without DPN and healthy controls	Significantly higher in the DPN group vs. T2DM and controls	TLR4 and TNF-α were potential biomarkers for DPN diagnosis. Higher levels were associated with increased DPN risk	Not reported
Zhu et al., 2017 [31]	Healthy controls	Higher in DPN compared to T2DM and controls	TLR4 and TNF-α positively correlated and caveolin-1 negatively correlated with DPN	None reported

**Figure 4:**
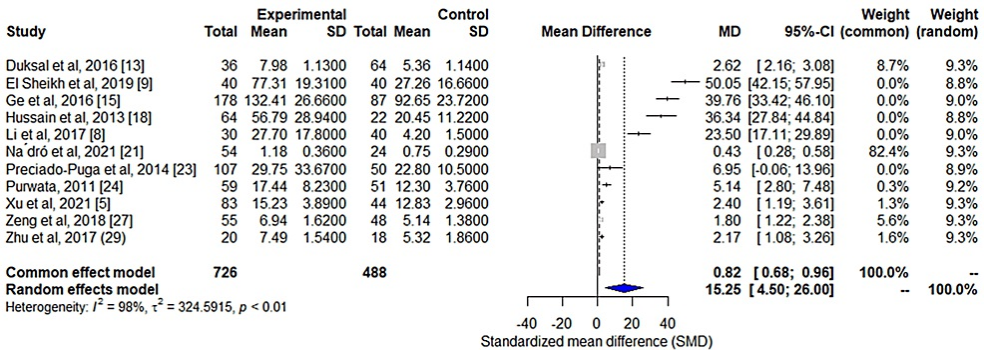
Forest plot of the included studies. Number of studies: k = 11; number of observations: o = 1,214; quantifying heterogeneity: tau2 = 324.5915 [155.1775; 1033.8814]; tau = 18.0164 [12.4570; 32.1540]; I^2^ = 98.1% [97.5%; 98.6%]; H = 7.30 [6.36; 8.38]; test of heterogeneity: Q = 532.81; degree of freedom = 10; p-value <0.0001; details of meta-analytical method: inverse variance method, restricted maximum-likelihood estimator for tau2, Q-profile method for confidence interval of tau2 and tau.

The results from the meta-analysis of 11 included studies revealed varying MDs and corresponding 95% CIs across different studies (Table 5). Notably, the forest plot (Figure 4) illustrated substantial heterogeneity among the studies, as indicated by a high I^2^ value of 98.1%. The heterogeneity was assessed by tau2 and tau, which were 324.5915 and 18.0164, respectively. These results, coupled with the Q-profile method, revealed the necessity for a more conservative strategy to accommodate the heterogeneity among the included studies.

**Table 5:** Summary of meta-analysis. MD: mean difference; CI: confidence interval; W: weight

	MD	95% CI	%W (common)	%W (random)
Duksal et al., 2016 [13]	2.62	2.1571; 3.0829	8.7	9.3
El Sheikh et al., 2019 [9]	50.05	42.1465; 57.9535	0.0	8.8
Ge et al., 2016 [15]	39.76	33.4211; 46.0989	0.0	9
Hussain et al., 2013 [18]	36.34	27.8399; 44.8401	0.0	8.8
Li et al., 2017 [8]	23.5	17.1135; 29.8865	0.0	9
Nádró et al., 2021 [21]	0.43	0.2794; 0.5806	82.4	9.3
Preciado-Puga et al., 2014 [23]	6.95	-0.0622; 13.9622	0.0	8.9
Purwata, 2011 [24]	5.14	2.8001; 7.4799	0.3	9.2
Xu et al., 2021 [5]	2.4	1.1895; 3.6105	1.3	9.3
Zeng et al., 2018 [27]	1.8	1.2206; 2.3794	5.6	9.3
Zhu et al., 2017 [29]	2.17	1.0774; 3.2626	1.6	9.3

Under the common-effect model, the pooled MD was 0.8210 (95% CI = 0.6843; 0.9576), with a z-value of 11.77 and a p-value less than 0.0001. The random-effect model showed a higher mean difference of 15.2464 (95% CI =4.4963; 25.9965), with a z-value of 2.78 and a p-value of 0.0054. The use of the random-effect model is justified due to the significant heterogeneity observed among the studies.

The forest plot provides a visual depiction of the effect sizes of individual studies, coupled with the combined effect size from the meta-analysis (Figure 4). It is obvious from the plot that the overall effect is positive, demonstrating increased TNF-α levels in DPN patients compared to diabetic individuals without neuropathy.

The sensitivity analysis was performed by eliminating one study at a time and estimating the overall MD for both fixed-effect and random-effect models. The overall effect sizes were consistent across varied exclusions, showing the robustness of the meta-analysis results. No single study appeared to have a disproportionate influence on the total effect size.

Discussion

We performed this systematic review and meta-analysis to assess the association between TNF-α and DPN risk in diabetic patients. We found 23 studies where serum TNP-α levels were studied in diabetic patients with peripheral neuropathy compared to patients without neuropathy. Among them, 19 studies showed an increase in TNF-α levels, and four studies showed no significant change in serum TNP-α levels in patients with DPN.

DPN is the most predominant neuropathic syndrome in diabetic mellitus patients. It can lead to severe neuropathic pain, ulceration, and lower limb amputation. The exact mechanism for the development of DPN is not fully understood but may include derangement in pathways related to hyperglycemia, dyslipidemia, microvascular complications caused by oxidative stress, neuronal inflammation, mitochondrial damage, and cell death [4]. Chronic low-grade inflammation is the major determinant for the development of DPN. There occurs activation of innate immune response, alteration in the insulin signaling pathway, insulin resistance, and mitochondrial and endoplasmic reticulum stress due to chronic low-grade inflammation. All these lead to the production of various kinases such as protein kinase C, jun-N terminal kinase, mitogen-activated protein kinase, and the release of various proinflammatory cytokines and interleukins such as 1b, 2, 6, 8, and TNP-α which play a vital role in the pathophysiology of DPN [32].

Numerous markers are used to predict the risk of developing DPN in patients with diabetes. These include macrophage-colony stimulating factor (CSF-1), monocyte chemoattractant protein-1 (MCP-1), vascular cell adhesion molecule-1, intercellular adhesion molecule-1 (ICAM-1), TNP-α, C-reactive protein (CRP), and E-selectin [33]. There is a significant alteration in innate immunity in diabetes patients. Toll-like receptor 4 (TLR4) receptors play an important role in recognizing the pathogen and initiating the immune response. Two significant pathways, namely, mitogen-activated protein kinase (MAPK) and nuclear factor kappa B (NF-κB), are activated by TLR4. They lead to the release of TNF-α and IL-6 and play a vital role in the regulation of neuronal plasticity [30].

A proinflammatory cytokine, TNP-α produced by monocytes and activated macrophages contributes significantly to the DPN pathophysiology. Patients with prolonged hyperglycemia have dysregulated TNF-α levels. In DPN patients, myelinated nerves show demyelination while the unmyelinated ones suffer axonal loss. T cells are activated due to immune response which produces TNP-α, mediating the inflammatory reactions. It causes oligodendrocyte toxicity leading to demyelination. The secretions of other cytokines such as IL-1β and IL-6 are increased from endothelial cells and monocytes that adds to damage in neurons. It also leads to hemodynamic abnormalities, endothelial dysfunctions and increased expression of various cell adhesion molecules in blood vessels, and decreased nitric oxide synthase activity in endothelial cells [9].

Anti-TNF-α medications may be evaluated as a viable method for controlling DPN. In DPN rats, the suppression of the TNF-α pathway utilizing a recombinant human TNF-α receptor-antibody fusion protein (rhTNFR:Fc) demonstrated recovery from nerve injury [34]. Other drugs such as infliximab, etanercept, sesamol, and rimonabant, which can either decrease or modify TNF-α levels, have shown promising benefits in DPN rats [35]. However, further study is necessary to explore the impact of anti-TNF-α therapy in DPN patients.

While this meta-analysis provides valuable insights, it is important to note some limitations, with the primary one being the heterogeneity among the studies. Differences in population demographics, study design, and diagnostic methods may impact the generalizability of our findings. Additional constraints include the fact that there are merely three prospective cohort studies examining the correlation between serum TNF-α levels and DPN in patients with diabetes mellitus. The majority of studies included in this meta-analysis were either case-control or cross-sectional studies which might lead to bias to a certain extent as all the confounding factors were not taken into consideration. Thus, there is a need for more prospective cohort studies to get a more accurate relation between serum TNF-α levels and the risk of DPN in diabetes patients. Another major limitation of this meta-analysis is that there is only one study that evaluated the association between serum TNF-α levels and the risk of DPN in type 1 diabetes mellitus.

## Conclusions

This systematic review and meta-analysis of diverse studies suggests a substantial relationship between higher TNF-α levels and DPN. The findings underline the potential function of TNF-α as a biomarker and contributor to diabetic neuropathy. Despite study variability, the systematic review provides a complete overview, emphasizing the need for more research to explain the processes of TNF-α in DPN and its therapeutic implications.
